# Inverted Nutcracker Syndrome: A Case of Persistent Hematuria and Pain in the Presence of a Left-Sided Inferior Vena Cava

**DOI:** 10.1100/tsw.2011.100

**Published:** 2011-05-05

**Authors:** Obi Ekwenna, Michael A. Gorin, Miguel Castellan, Victor Casillas, Gaetano Ciancio

**Affiliations:** ^1^ Department of Urology, Division of Transplantation, University of Miami School of Medicine, Miami, Florida, USA; ^2^ Department of Radiology, Division of Transplantation, University of Miami School of Medicine, Miami, Florida, USA; ^3^ Department of Surgery, Division of Transplantation, University of Miami School of Medicine, Miami, Florida, USA

**Keywords:** renal autotransplantation, Nutcracker syndrome, anatomic anomalies, inferior vena cava

## Abstract

Nutcracker syndrome is described as the symptomatic compression of left renal vein between the aorta and the superior mesenteric artery, resulting in outflow congestion of the left kidney. We present the case of a 51-year-old male with a left-sided inferior vena cava, resulting in compression of the right renal vein by the superior mesenteric artery. Secondary to this anatomic anomaly, the patient experienced a many-year history of flank pain and intermittent gross hematuria. We have termed this unusual anatomic finding and its associated symptoms as the “inverted nutcracker syndrome”, and describe its successful management with nephrectomy and autotransplantation.

## INTRODUCTION

Nutcracker phenomenon (NCP) refers to the anatomic compression of the left renal vein (LRV) between the superior mesenteric artery (SMA) and abdominal aorta, resulting in impedance of outflow from the LRV[1]. NCP should be differentiated from nutcracker syndrome (NCS), which describes the clinical symptoms associated with demonstrable nutcracker anatomic features[2]. Although rare, NCS is well described in the literature[3,4,5,6]. This syndrome results in renal venous hypertension and is characterized by hematuria, development of collateral vessels, pain, chronic fatigue syndrome, proteinuria, and gastrointestinal symptoms.

NCS is usually associated with the left kidney, except for a single case of a right-sided NCP secondary to compression of the right renal vein (RRV), inferior vena cava (IVC), and right gonadal veins by the presence of gravid uterus[7]. We present the first case of right-sided nutcracker syndrome resulting from a left-sided IVC anomaly, and describe the successful management with right nephrectomy and autotransplantation.

## CASE REPORT

### Patient

A 51-year-old male with an otherwise unremarkable past medical history presented to our care as a quaternary referral with a 13-year history of gross hematuria requiring multiple blood transfusions. The episodes of hematuria were associated with chronic fatigue and right flank pain/groin pain. The patient's symptoms were present intermittently and associated with strenuous physical activity.

Over the preceding 13 years, the patient underwent an extensive hematuria workup[8] at several institutions in the U.S. Multiple imaging studies, including renal ultrasound and contrast-enhanced computed tomography (CT) of the abdomen and pelvis, were interpreted as normal anatomy without pathologic findings, thus leading to the ultimate diagnosis of lion pain-hematuria syndrome, a diagnosis of exclusion.

On initial consultation, physical examination was unremarkable, including no evidence of external varicosities or varicocele. The patient's body mass index was 22.1 kg/m$^2$. Urine analysis showed microscopic hematuria and mild proteinuria; all other workup for hematuria including a cystoscopy and cytology were negative. Serum analysis found a hemoglobin of 12 g/dL and a normal coagulation panel.

Careful rereview of a multiphase helical CT scan revealed orthotopic bilateral renal units. Evaluation of the vasculature demonstrated the SMA entrapped the RRV as it crossed over the aorta to insert into a left-sided IVC (Figs. 1 and 2). In addition, the right gonadal and adrenal veins drained into the RRV (Fig. 2). No evidence of venous collaterals was present. Given this anomalous anatomy, it was felt that autotransplantation of the right kidney would offer the best chance of surgical correction of symptoms.

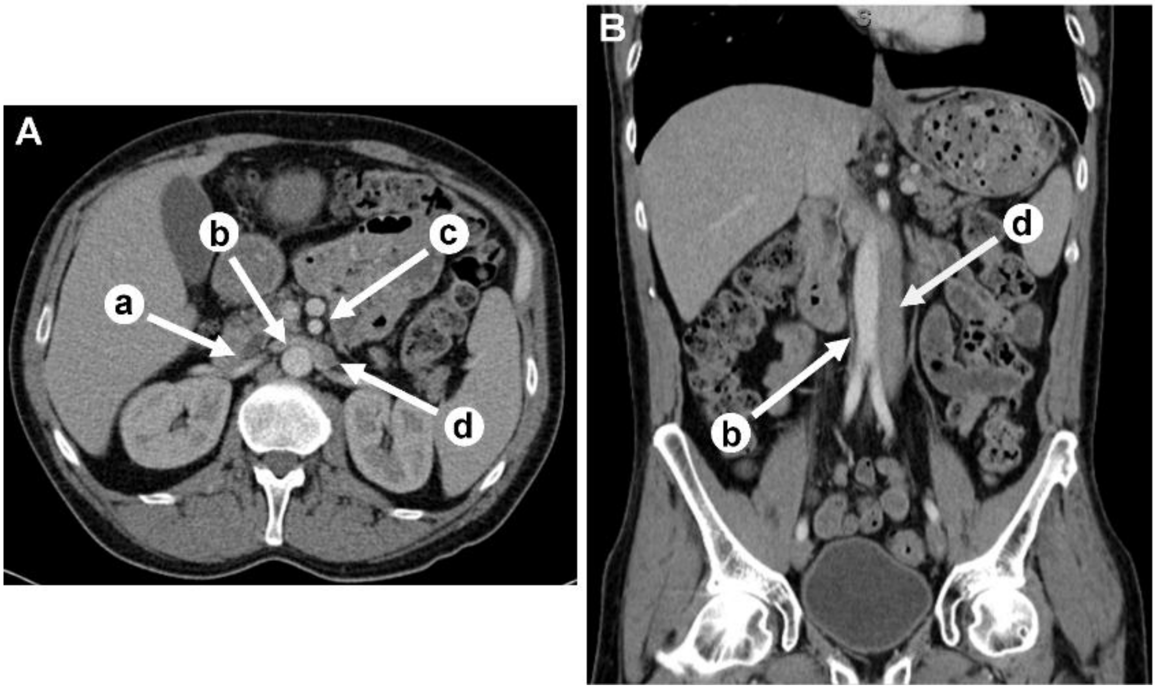

**FIGURE 1.** (A) Axial CT of the abdomen with contrast and (B) coronal reconstruction demonstrating the inverted nutcracker syndrome. a = Right renal vein, b = right-sided aorta, c = superior mesenteric artery, d = left-sided vena cava.

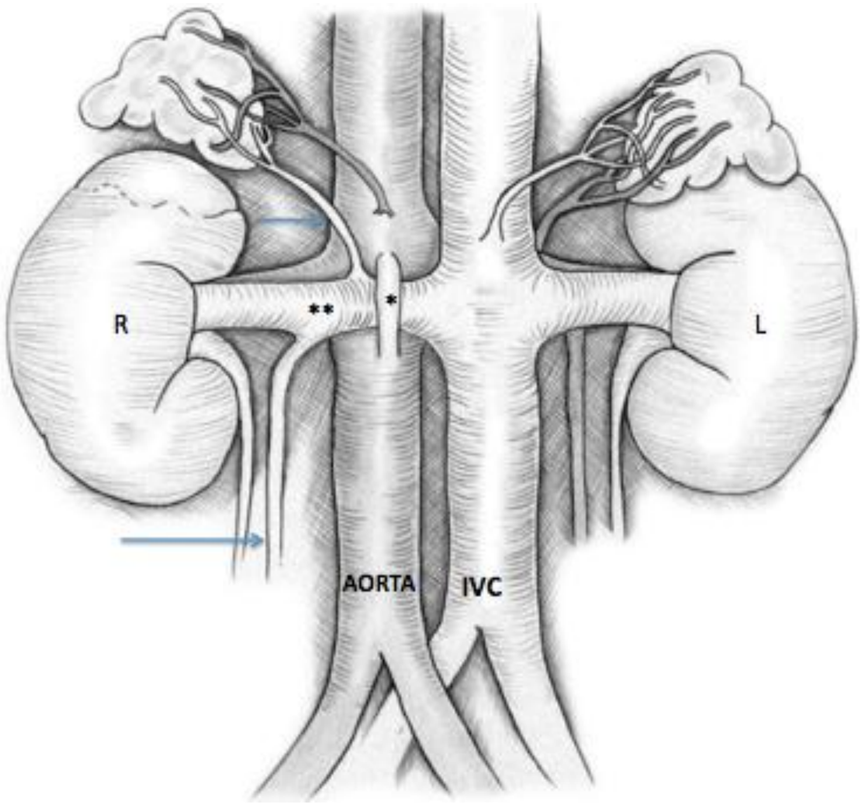

**FIGURE 2.** Schematic illustration of the patient's anomalous anatomy. Short arrow = right adrenal vein, long arrow = right gonadal vein, ** = right renal vein, * = superior mesenteric artery.

## Surgical Technique

A right, open-donor nephrectomy was performed with the standard retroperitoneal flank approach[9]. Upon dissection of the right renal hilum, the RRV was seen joining the left IVC; the right adrenal and gonadal veins were draining directly into the RRV (Fig. 2). There were several small collaterals, which were ligated and divided. The main artery and vein were then isolated in the usual fashion. The right renal artery (RRA) was ligated along the aorta, and a Satinsky clamp was used to clamp the IVC and transect the RRV. The ureter was isolated and divided. Next, the kidney was removed and a renal biopsy performed. A Gibson incision was made on the left side to isolate the external iliac vessels. The RRA and RRV were anastomosed to the external iliac artery and vein, respectively. Extravesical ureteroneocystostomy was performed in a modified Lich-Gregoir technique. The incision was closed after a Jackson Pratt drain was placed.

## Perioperative Period and Follow-Up

On postoperative day 1, a duplex ultrasound of the transplanted kidney was obtained, which demonstrated flow velocities in the normal range for a transplanted kidney. Pathologic evaluation of the renal biopsy showed no abnormality. The patient was discharged on postoperative day 6 and recovered without complication. At 6 weeks follow-up, the patient reported no episodes of gross hematuria and a minor analgesic requirement for pain. A urinalysis was normal with no evidence of microscopic hematuria or proteinuria.

## DISCUSSION

In 1972, De Schepper[10] first described NCS as the compression of the LRV between the aorta and the SMA with symptomatic presentation. Symptoms associated with NCS include gross or microscopic hematuria, orthostatic proteinuria, flank and abdominal pain, varicocele, pelvic congestion, and chronic fatigue. To our knowledge, we report the first case of NCS involving the RRV with a left-sided IVC. We have termed this unusual anatomic finding the "inverted nutcracker syndrome" (INCS).

Congenital IVC anomalies are rare findings and have been reported since the earliest anatomic descriptions dating back to 1700s. The formation of the IVC occurs in the 6–8$^{th}$ week of gestation from three-paired cardinal veins: the posterior cardinal, subcardinal, and supracardinal veins. In normal development, these vessels regress and become confluent as the singular IVC. Deviation from normal development may result in a variety of anomalous anatomic configurations[11]. The formation of the left-sided IVC results from the regression of the right supracardinal vein with persistence of the left supracardinal vein. The prevalence of this configuration is 0.2–0.5%. In the presented case, the RRV crosses anterior to the aorta to drain into the left-sided IVC. *INCS is, therefore, the symptomatic consequence of entrapment of the RRV between the SMA and aorta.*

Risk factors for NCS remain unknown[3,5,6,12,13]. However, there appears to be a slightly higher incidence in females. The age at presentation is variable; cases of child and adult manifestations have been reported in the literature. Children are usually asymptomatic[14]. In our patient, his symptoms, which included hematuria, pain, and proteinuria, were aggravated by physical activity. The most common reported symptom of NCS is hematuria, which is attributed to development of collateral shunts in the collecting system secondary to elevated venous pressure. Gross hematuria or microhematuria ensues with or without significant anemia. Various authors have reported cases requiring blood transfusion as in our case[4,5,7,12,15,16]. Pain is the second most common symptom. This may be part of the gonadal vein syndrome, which is characterized by abdominal or flank pain that radiates to the posteromedial thigh and buttocks. Physical activity may exacerbate this pain. Flank pain is a result of ureteral colic, from blood clots passing down the ureter.

Shin and Lee[12] associated NCS with chronic fatigue syndrome, correlating with high LRV-IVC pressure gradients. The proteinuria observed is a result of lysis of red blood cells under high pressure. The exact mechanism and pathophysiology[3] of NCS is not understood, however, the general consensus is that abnormal branching of the SMA from the aorta at an acute angle with an initial steep caudal descent causes compression of the LRV, or inversely RRV in the case of a left IVC.

NCS is a difficult diagnosis to make and is often a diagnosis of exclusion. In our case, the diagnosis eluded many clinicians for over 13 years. Some authors[4] have proposed extensive workup prior to diagnosis of NCS, including IVC and RV venography, venous pressure manometry, cystoureteroscopy, MR angiography, and CT urogram, among other tests. We believe that thorough evaluation of the common causes of hematuria be ruled out before making the diagnosis of NCS. Venography may be not necessary given the broad utility of multiphase helical CT scan.

Conservative management is reserved for mild hematuria and associated pain. Surgical intervention is indicated for severe and persistent gross hematuria and unrelenting pain. Various surgical approaches have been described including nephropexy, LRV bypass, endovascular stenting, SMA transposition, and transposition of the LRV[6,13]. In addition, autotransplantation is an alternative treatment with excellent outcomes and relatively less morbidity[6,17]. The patient presented here underwent a successful autotransplantation without complications. In follow-up, the patient had no evidence of hematuria with resolving pain.

## CONCLUSION

INCS is an extremely rare anatomic variant. A high index of suspicion should be raised for NCS when unilateral hematuria and pain persists with a negative hematuria workup. Here we present the first reported case of INCS. Autotransplantation proved to be a technically feasible treatment for this patient. Meticulous surgical planning and medial vascular dissection was essential for a favorable outcome.
